# Assaying sensory ciliopathies using calcium biosensor expression in zebrafish ciliated olfactory neurons

**DOI:** 10.1186/s13630-018-0056-1

**Published:** 2018-03-15

**Authors:** Judith G. M. Bergboer, Cameron Wyatt, Christina Austin-Tse, Emre Yaksi, Iain A. Drummond

**Affiliations:** 1 Nephrology Division, Department of Medicine, Massachusetts General Hospital, 149 13th Street, Charlestown, MA 02129 USA; 2 Department of Genetics, Harvard Medical School, 25 Shattuck Street, Boston, MA 02115 USA; 3 Neuroelectronics Research Flanders, Kapeldreef 75, 3001 Louvain, Belgium; 4 Centre for Neural Computation, Kavli Institute for Systems Neuroscience, Olav Kyrres Gate 9, 7030 Trondheim, Norway

**Keywords:** Cilia, Calcium imaging, Zebrafish, GCaMP5, Olfactory sensory neurons, *oval/ift88*, *ift172*

## Abstract

**Background:**

Primary cilia mediate signal transduction by acting as an organizing scaffold for receptors, signalling proteins and ion channels. Ciliated olfactory sensory neurons (OSNs) organize olfactory receptors and ion channels on cilia and generate a calcium influx as a primary signal in odourant detection. In the zebrafish olfactory placode, ciliated OSNs and microvillus OSNs constitute the major OSN cell types with distinct odourant sensitivity.

**Methods:**

Using transgenic expression of the calcium biosensor GCaMP5 in OSNs, we analysed sensory cilia-dependent odour responses in live zebrafish, at individual cell resolution. *oval/ift88* mutant and *ift172* knockdown zebrafish were compared with wild-type siblings to establish ciliated OSN sensitivity to different classes of odourants.

**Results:**

*oval/ift88* mutant and *ift172* knockdown zebrafish showed fewer and severely shortened OSN cilia without a reduction in OSN number. The fraction of responding OSNs and response amplitudes to bile acids and food odour, both sensed by ciliated OSNs, were significantly reduced in *ift88* mutants and *ift172*-deficient embryos, while the amino acids responses were not significantly changed.

**Conclusions:**

Our approach presents a quantitative model for studying sensory cilia signalling using zebrafish OSNs. Our results also implicate *ift172*-deficiency as a novel cause of hyposmia, a reduced sense of smell, highlighting the value of directly assaying sensory cilia signalling in vivo and supporting the idea that hyposmia can be used as a diagnostic indicator of ciliopathies.

**Electronic supplementary material:**

The online version of this article (10.1186/s13630-018-0056-1) contains supplementary material, which is available to authorized users.

## Background

Sensory function and sensory input elicits an appropriate response to external stimuli. Several sensory systems, including vision and olfaction, rely on cilia-mediated sensory transduction [1, 2]. Ciliopathies, a class of complex genetic syndromes caused by mutations in genes encoding cilia proteins, underscore the importance of cilia. Clinical features include renal cysts, laterality defects, cognitive impairment, retinal degeneration and obesity [3, 4]. Anosmia and hyposmia, absence or reduction of olfaction, respectively, have also been shown to be a component of the phenotype in human syndromic ciliopathy patients, including the Bardet–Biedl syndrome (BBS), as well as several mouse models for ciliopathies [5–9]. Recent reports indicate that in addition to mutations in genes that encode cilia transition zone and vesicular transport proteins, syndromic ciliopathy may be caused by mutation in genes encoding intraflagellar transport proteins (IFTs) that are directly involved in building the axoneme [10–12].

In vertebrates, odourants are detected by olfactory sensory neurons (OSNs) located in the olfactory epithelium (OE) in the nasal cavity. In ciliated OSNs, dendrites terminate in a sensory knob from which sensory cilia arise. These cilia are enriched for all necessary components for olfactory signal transduction. In zebrafish, each ciliated OSN expresses one of approximately 140 different G-protein coupled olfactory receptors (ORs) [13–15]. Binding of odourants to the OR activates the olfaction-specific G-protein G_α/olf_, which activates adenyl cyclase III to generate the second messenger cyclic adenosine monophosphate (cAMP). Elevated levels of cAMP activate the olfactory cyclic nucleotide-gated channels, leading to Na^+^ and Ca^2+^ influx and Cl^−^ efflux, triggering depolarization and propagation of action potentials [16]. In zebrafish, the two most abundant types of OSNs are the olfactory marker protein (Omp)-positive ciliated OSNs and the transient receptor potential channel C2 (TrpC2)-positive microvillus OSNs, which have been shown to preferentially respond either to bile acids or amino acids, respectively [17, 18]. With OSN cilia directly exposed to the larval environment and visually accessible in transparent larvae, the zebrafish provides a vertebrate system to study sensory cilia function in vivo, complementing approaches developed in Chlamydomonas and *C. elegans* [19, 20].

In this study, we combined live imaging of neuronal calcium activity induced by odourants with genetic manipulation in zebrafish to study the effect of cilia protein deficiency on sensory cilia function. We used *Tg(elavl3:GCaMP5)* transgenic zebrafish expressing the calcium indicator GCaMP5 under the *elavl3* (formerly known as HuC) promoter in nearly all neurons including the ciliated OSNs [21, 22]. In addition, we generated a *Tg(omp:GCaMP6)* line, to specifically monitor the responses of the ciliated OSNs. These lines enabled recording of cilia-dependent OSN functional activity with single cell resolution after stimulation with different classes of odourants in vivo. Deficiencies in intraflagellar transport (*ift*) genes essential for ciliogenesis (*oval/ift88* and *ift172*) severely affected OSN cilia length and number. We show that deficiency of either *ift* significantly reduced OSN responses to bile acids while the response to amino acids was not significantly changed. Our results show that sensory cilia-dependent signalling can be measured and quantified in living zebrafish. In addition, we show that *ift172* deficiency is a novel cause of hyposmia.

## Methods

### Zebrafish strains and maintenance

Zebrafish (*Danio rerio*) were maintained according to standard procedures [23] and experiments were performed in accordance with approved animal care guidelines under MGH IACUC (Boston) and EU (Leuven) licenses. These lines were previously published: *Tg(trpc2:Venus), Tg(omp:gal4)* [17], *Tg(elavl3:GCaMP5) nacre*^−/−^ [24] and *oval*/*ift88*^−*/*−^ [25]. *Tg(omp:mCherry)* and *Tg(omp:GCaMP6f)* were generated by standard Tol2-mediated transposition approach [26]. Vectors for transgenesis were generated by multisite Gateway recombination of the following vectors: pENTR-5′ containing the *omp* promoter, pDestTol2CG2, pME-mCherry or pME-GCaMP6f and p3E-polyA [27]. The *omp* promoter was amplified by PCR using the OMP:Venus vector as a template [17], for primer sequences see Additional file 1: Table S1. For ISH and staining of pigmented embryos, embryos were kept in E3 with 0.003% 1-phenyl-2-thiourea (PTU) (Sigma-Aldrich) from 24 hpf onwards.

### Morpholino knockdown

The exon1d splice donor antisense MO was used to target *ift172*, sequence 5′-TTTACCTGAGGCGTTAAAAGAGTCT-3′. As a control MO 5′-CTCGACCGTCCATTTAAGTCAAATA-3′ was used. MO oligonucleotides were diluted 0.25 mM in 100 mMKCl, 10 mM HEPES and 0.1% phenol red (Sigma-Aldrich, St Louis, MO). 4.6 nl was injected into one- to four-cell stage embryos. To verify the MO-induced splicing defects in the *ift172* morphants, nested RT-PCR was performed on total RNA extracted from individual MO-injected embryos. For primer sequences, see Additional file 1: Table S1.

### Transmission electron microscopy

Zebrafish embryos were prepared for TEM by previously published protocols [28].

### Cep290 antibody generation

The Cep290 C terminal antibody was developed at Rockland Immunochemicals by immunizing rabbits with a maltose binding protein-Danio rerio Cep290 fusion protein (Cep290 amino acids 2220–2396). Antibodies were affinity purified using 6×His-Cep290 (amino acids 2220–2396) on an NTA Ni column. Staining of basal body domains in Kupffer’s vesicle (the zebrafish node) was eliminated by preincubation of the antibodies with free antigen (Additional file 1: Fig S7), demonstrating the specificity of Cep290 immunoreactivity.

### Immunofluorescent imaging

Embryos were fixed in dents fixative (80% MeOH, 20% DMSO) overnight at 4 °C or 4% paraformaldehyde (PFA) for 2 h and permeabilized in methanol for 7 min at RT. After rehydration, embryos were blocked in PBST with 10% normal goat serum at RT for 2 h, samples were incubated with primary antibodies overnight at 4 °C. Used dilutions: rabbit anti-IFT88 (1:400, gift from Brain Perkins), chicken anti-GFP (1:500, Invitrogen), mouse anti-acetylated α-tubulin (6-11B-1 1:400, Sigma), rabbit anti-cep290 (1:200), rabbit anti-G_α/olf_ (1:1000, Santa Cruz), Alexa 488- or 546-conjugated goat anti-rabbit, goat anti-chicken or goat anti-mouse secondary antibodies (1:800, Invitrogen). For imaging, embryos were cleared in 53% benzyl alcohol, 45% glycerol and 2% *N*-propyl gallate. Two-colour *z* series were acquired on a Zeiss LSM5 Pascal confocal microscope via sequential laser excitation. Images were deconvolved using the Huygens Essential program (Scientific Volume Imaging, Hilversum, Netherlands) and processed using Adobe Photoshop software. For sensory cilia length measurements, the central sensory placodes (excluding peripheral motile cilia) of anti-G_α/olf_-stained embryos were imaged in *Z*-stacks in a dorsal–ventral orientation on an upright Zeiss LSM5 Pascal confocal microscope. Deconvolved confocal stacks were analysed in ImageJ using the line tool to measure cilia length orthogonal to the *Z* axis. Any foreshortening due to cilia angle may lead to underestimates of the absolute length, however, since this applies to both mutant and wild-type, relative length measurements would not be affected. Fluorescent intensity measurements of anti-ift88 immunostaining were made using ImageJ to measure regions of interest encompassing olfactory placodes and correct for ROI area and background fluorescence.

### In vivo confocal imaging

2.5-dpf-old, size-matched transgenic embryos were mounted and two-colour *z* series were acquired on a Zeiss LSM5 Pascal confocal microscope system using a water-dipping lens via sequential laser excitation.

### RNA in situ *hybridization*

Whole mount in situ *hybridization* was performed as previously described [29], without the ProtK treatment to preserve the superficial localized olfactory epithelium. The *omp* probe was produced as described [30]. Stained, 4% PFA fixed embryos were transferred into PBS:glycerol (1:1) and imaged on a Leica MZ12 microscope equipped with a Spot Image digital camera.

### Preparation of odour stimuli

Frozen stock solutions (10 mM) of amino acids, bile acids or nucleotides of the highest available purity (Fluka; Sigma-Aldrich) were diluted to a final concentration of 10 µM per component. Amino acids mixture contained arginine, asparagine, aspartic acid, alanine, phenylalanine, histidine and methionine. Bile acid mixture consisted of glycocholic acid, taurodeoxycholic acid, taurochenodeoxycholic acid and taurocholic acid. Nucleotides mixture contained inosine monophosphate and adenosine monophosphate. Artificial fish water (AFW, 1.2 g of Instant Ocean sea salt per 20 l of water) was used as water control. Food odour was prepared by vortexing 0.5 g of ZM000 fish food in 50 ml of AFW and then incubating for 1 h at RT. To remove undissolved particles, the saturated mixture was passed through filter paper.

### Olfactory assay

2.5-dpf-old healthy, size-matched transgenic zebrafish were mounted in 1.5% low melting agarose. The nose region was cleared to provide access to the nostrils. Under a continuous flow of AFW (100 ml/h), each of three odourants (amino acids, bile acids, or nucleotides) were delivered by a computer-controlled HPLC valve (Rheodyne). Odours were delivered three times in a randomized order, separated by 1–2 min to exclude sensory adaptation. Imaging of the OE was performed with a two-photon laser scanning system (LSM 7 MP upright with 20× water immersion objective, Zeiss) at ~ 3 Hz. A mode-locked Ti:Sapphire laser tuned to 920 nm was used for excitation. Images were acquired using ZEN software (Zeiss).

### Data analysis

Odour-response data analyses were performed using custom scripts written in MATLAB (Mathworks) [31]. Images were aligned, and for each trial, the change in fluorescence (Δ*F*/*F*, DFF) relative to a control trial was calculated for each pixel, yielding a series of DFF frames. Pixels belonging to individual neurons were identified [32]. Automatically identified neurons were always manually confirmed. The relative change in fluorescence (DFF) was calculated for each neuron over time. Fluorescence time courses for each neuron were averaged across multiple stimulus trials. From these per neuron per frame DFF values, all subsequent analyses were performed using standard MATLAB functions. All odours were presented to larvae in triplicate (technical replicates) and measurements were made on up to ten different embryos from each of six different clutches of embryos (biological replicates). To minimize technical and biological variation, only datasets containing both control and affected embryos scanned on the same days and from the same clutches were included in the analyses. For odour measurements, we collected data from 80 to 100 OSNs per larvae and used up to ten different larvae and repeated measurements on six different clutches of embryos. Responding OSNs were defined as those exhibiting an odour evoked response deviating > ± 2 SD from the baseline. Baseline was calculated individually for all cells based on their water response (negative odour control). No significant difference was observed between water responses for wild-type and mutant OSNs. DFF values were either plotted for the responding OSNs only (Figs. 3, 4) or with all OSNs included (Fig. 2 and Additional file 1: Fig. S4). For the responding OSNs only: DFF values were multiplied by number of responding cells to a specific odour and divided by the total number of cells which represents the DFF for each cell relative to their contribution to the response of the entire olfactory epithelium.

### Statistical analysis

Mann–Whitney *U* test was performed to compare response strengths to individual stimuli compared to water control (Fig. 2), to compare response strengths and responding cell counts to similar stimuli between groups (Figs. 3, 4). Student’s *t* test was used to compare cell numbers and OSN sensory cilia length for which G_α/olf_ staining was used as a marker. Cilia length was measured using ImageJ. *P* < 0.05 was considered statistically significant.

## Results

### Cilia and ciliated OSNs in the zebrafish olfactory placode

**Fig. 1:**
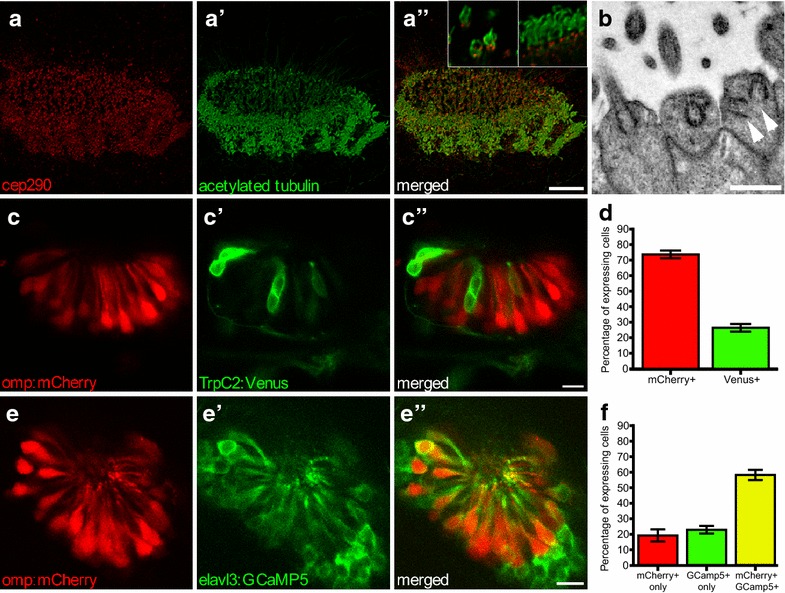
Olfactory epithelium (OE) in 2.5-dpf zebrafish. Multi-ciliated motile cilia are present at the border, ciliated OSNs in the centre; maximum intensity projection. **a** Basal bodies stained with anti-cep290 (red), **a’** cilia stained with anti-acetylated tubulin (green), **a”** merged image. Inset in **a”** closeup of sensory (left) and motile (right) cilia showing basal body staining of anti-Cep290. **b** EM image of dendritic knob of ciliated zebrafish OSNs containing multiple basal bodies (arrowheads). **c** *Tg(omp:mCherry,trpc2:Venus)* zebrafish with ciliated Omp-positive OSNs (red) and **c’** microvillus TrpC2-positive OSNs (green), **c”** merged image; single confocal section. **d** Quantification of C, *N* = 5 fish. **e** *Tg(omp:mCherry,elavl3:GCaMP5)* zebrafish show overlap between ciliated Omp-positive OSNs (red) and **e’** GCaMP5-positive OSNs (green) OSNs, **e”** merged single confocal section. **f** Quantification of E, *N* = 6 fish. Bars represent mean and SEM. Scale bar is 10 µm, except **b** bar is 1 µm

Two types of ciliated cells are present in the zebrafish olfactory placode: motile and sensory (Fig. 1a) [33]. At the OE border motile multi-ciliated cells drive fluid across ciliated sensory nerve endings of OSNs in the centre (Additional file 1: Movie S1) [33, 34]. Similar to mammalian OSNs, zebrafish ciliated OSNs harbour multiple basal bodies in their olfactory knob (Fig. 1b) to support multiple cilia per neuron [33]. Next to ciliated Omp-positive OSNs, microvillus TprC2 expressing OSNs are present in the OE [17]. At 2.5 days post fertilization (dpf), *Tg(omp:mCherry,trpc2:Venus)* zebrafish OSNs showed mutually exclusive expression of Omp or TrpC2 with the majority being ciliated, Omp positive, OSNs (74%) and 26% were TrpC2 positive, microvillus OSNs (Fig. 1c, d). To assay both ciliated and non-ciliated cell responses to odourants, we used *Tg(elavl3:GCaMP5)* zebrafish which express the calcium indicator GCaMP5 pan-neuronally, including the OSNs. At 2.5 dpf *Tg(elavl3:GCaMP5,omp:mCherry)* zebrafish revealed an overlap of 58% between mCherry and GCaMP5-positive OSNs, while 23% of the cells were GCaMP5 positive only (*n* = 6 fish, Fig. 1e, f). Considering *omp:mCherry*-positive cells alone (ciliated OSNs), on average 76% of the cells were also GCaMP5 positive, confirming expression of GCaMP5 in the majority of ciliated OSNs.

### Odour detection in 2.5 dpf *Tg(elavl3:GCaMP5)* zebrafish

**Fig. 2:**
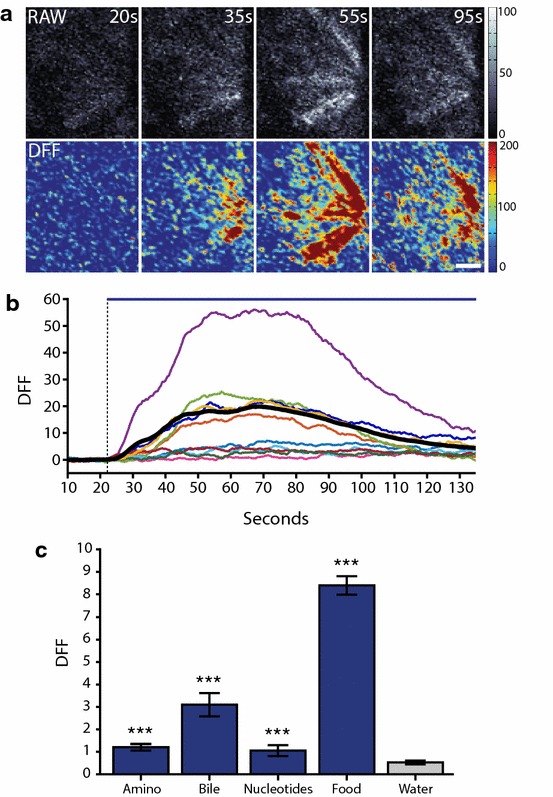
Odour detection in OSNs of wild-type *Tg(elavl3:GCaMP5)* at 2.5 dpf. **a** Frames of food odour response time course displayed as RAW image (top row) and DFF (change in fluorescence over baseline fluorescence; Δ*F*/*F*, DFF; bottom row). **b** Example traces of individual OSNs responses in time after addition of food odour, dashed line represents odour arrival at olfactory epithelium and solid line represents duration of odour delivery. Coloured lines represent individual example OSN responses, thick black line is mean of all OSNs (*N* = 382 OSNs). Data acquisition speed was 2 Hz. **c** Mean OSN-response amplitude to different odour classes used in this study compared to the water control (*N* = 4 fish, 382 OSNs, ***P < 0.001, Mann–Whitney *U* test). Bars represent mean and SEM. Scale bar is 10 µm

To establish OSN activity as a read-out for sensory cilia function in vivo, we tested 2.5 dpf wild-type *Tg(elavl3:GCaMP5)* zebrafish for OSN calcium responses to three different odourant classes: amino acids, bile acids, or nucleotides. These distinct classes are known to activate different spatial domains within the olfactory bulb [35], and thus suggested to stimulate ORs on different OSN subtypes [17]. As a positive control, we used food odour, containing molecules from all classes, activating a substantial proportion of OSNs. As expected, food odour stimulation produced robust, widespread calcium responses from OSNs (Fig. 2a, b, Additional file 1: Movie S2), while the specific odourant classes showed more confined responses. Analysis of 382 single OSNs (*N* = 4 fish) revealed that all odourants evoked significantly increased response amplitudes compared to water control (*P* < 0.001 Mann–Whitney *U* test, Fig. 2c), demonstrating that *Tg(elavl3:GCaMP5)* can be used for live, quantitative detection of sensory cilia signalling in zebrafish.

### OSN cilia defects in ift-deficient olfactory placodes

**Fig. 3:**
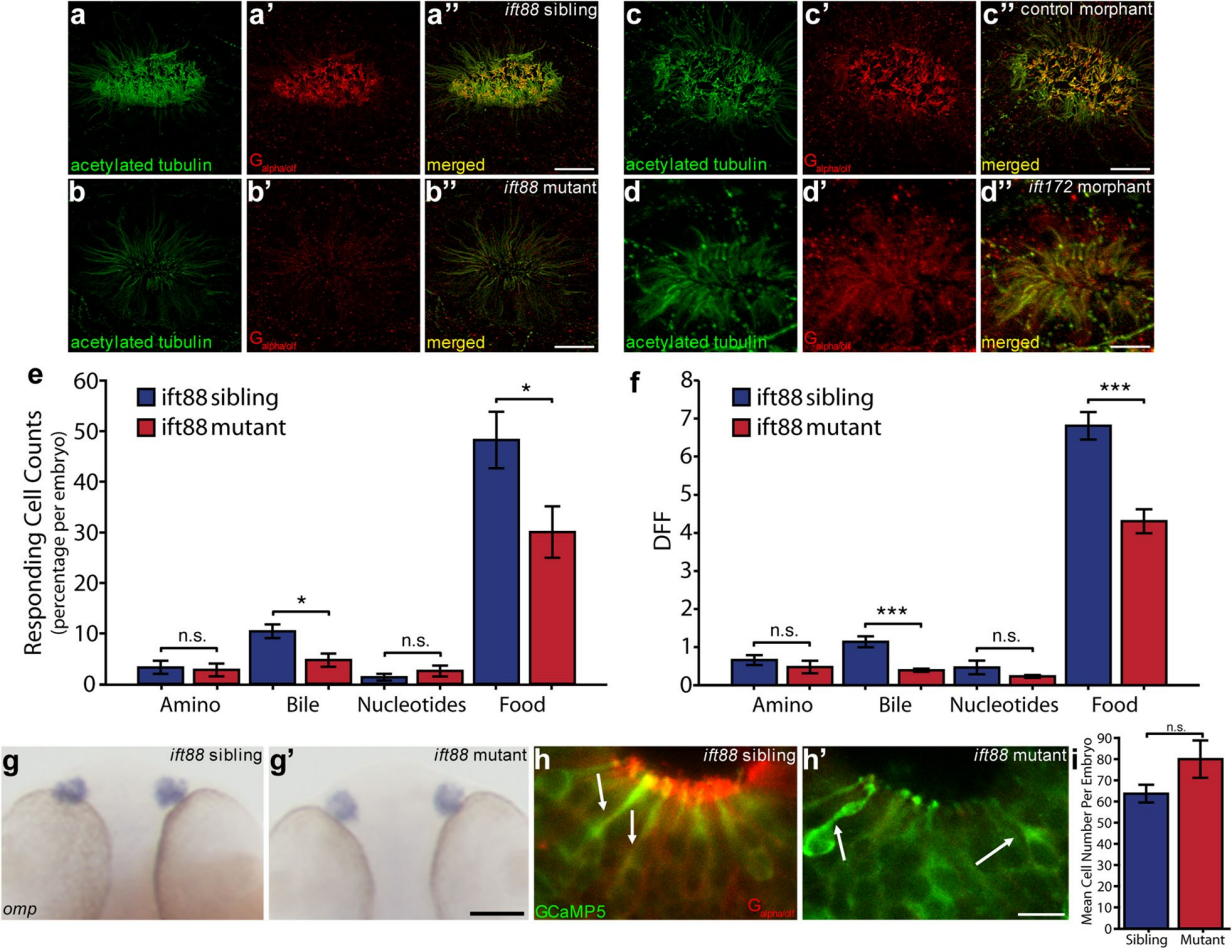
Cilia defects have functional effects in OSNs in Ift-deficient zebrafish at 2.5 dpf. **a** Anti-acetylated tubulin (green) stains all cilia axonemes in wildtype embryos as well as neuronal processes projecting away from the olfactory placode.** a’** Anti-Ga/olf (red) is a marker for OSN cilia.** a”** Merged image of** a** and** a’**.** b*** ift88 mutant* homozygotes show loss of acetylated tubulin positive axonemes and** b’** loss of anti-Ga/olf staining.** b”** Merged image of** b** and** b’**.** c** Control morpholino injected olfactory placode stained with anti-acetylated tubulin and** c’** anti-Ga/olf.** c”** Merged image of** c** and** c’**.** d*** ift172* morphant olfactory placodes show loss of anti-acetylated tubulin staining and** d’** loss of anti-Ga/olf staining.** d”** Merged image of** d** and** d’**. Remaining anti-acetylated tubulin staining is restricted to neuronal processes [67] (**b**,** d**). For quantification see Table 1.** e** Significant change in percentage of odour responding OSNs per embryo after addition of bile acids and food odour in* Tg(elavl3:GCaMP5) ift88 *mutant OSNs compared to sibling OSNs (*N* = 637 OSNs, 10 fish for sibling and *N* = 720 OSNs, 9 fish for mutant). **f** Significantly reduced response amplitude to bile acids and food odour in* Tg(elavl3:GCaMP5) ift88* mutant responding OSNs compared to sibling responding OSNs.** g** Whole mount in situ hybridization using an* omp* probe demonstrates clear* omp* expression in the OE in both wild type siblings** g** and** g’*** ift88* mutants and siblings at 2 dpf. (For other time points see Additional file 1: Fig. S6A).** h** Ciliated OSNs stained with anti-GFP (green) and anti-Ga/olf (red) present in both** h** sibling and** h’*** ift88* mutant, based on cell shape (arrows) in* Tg(elavl3:GCaMP5)* fish.** i** No difference in number of GCaMP5 positive OSNs per fish (*P* =  0.10). Bars represent mean and SEM (*P < 0.05, **P < 0.01, ***P < 0.001 Mann–Whitney *U* test for **e **and** f**, student’s *t* test for** i**. Scale bar is 10 μm, except panel **g** bar is 100 μm

To establish the role of Ift proteins in olfactory sensory ciliogenesis, we analysed the *oval* mutant (*ovl*^*tz288b*^), carrying a nonsense mutation in the *intraflagellar transport protein 88* (*ift88*) gene (L260X) [36]. In a separate set of experiments, we knocked down another *ift* gene, *ift172,* using an exon 1 splice donor blocking antisense morpholino (*ift172* morphants). Importantly, the *ift172* morphant phenocopied the *ift172* mutant phenotype [37]. In the *ift88* mutant, we confirmed a 99% reduction of the Ift88 immunofluorescence signal in the OE (*N* = 3 fish, Additional file 1: Fig. S1). *ift172* knockdown was confirmed by RT-PCR (Additional file 1: Fig. S2). Anti-G_α/olf_ immunofluorescence, a marker of OSN cilia, revealed a 77% reduction in the number of OSN cilia in *ift88* mutant OSNs and, in OSNs that still had cilia, a 46% reduction in cilia length compared to wild-type siblings (Fig. 3a, b, Additional file 1: Fig. S3, Table 1). Also, *ift172* morphants showed a 66% reduction in cilia number and a 43% shortening of remaining cilia compared to control morphants (Fig. 3c, d, Table 1). These results indicate that zygotic Ift-deficiency markedly reduces OSN cilia number and length at 2.5 dpf but does not completely eliminate ciliogenesis.

**Table 1:** Effect of Ift-deficiency on OSN cilia number and length

	N	Number of OSN cilia		*P* value	Length of OSN cilia (µm)^a^		*P* value
		Average	SD		Average	SD	
*ift88* sibling	3	109	24.6	0.0052	1.53	0.34	1.61E−20
*ift88* mutant	3	25.0	9.2		0.82	0.37	
control morphant	3	112	11.7	0.0035	2.03	0.54	2.73E−25
*ift172* morphant	2	37.5	3.5		1.15	0.51	

^a^Based on all or 50 OSN cilia per fish

### *ift88* mutant embryos show reduced cilia-dependent OSN activity

OSN responses to odourants were examined in 2.5 dpf *Tg(elavl3:GCaMP5) ift88* mutant zebrafish (720 OSNs, *N* = 9 fish) and compared to wild-type siblings (637 OSNs, *N* = 10 fish) (Fig. 3e, f). To control for any mechanosensory responses to potential change in rate of flow during valve switching for odour delivery, odour-responsive OSNs were first defined as the cells showing an odour evoked response exceeding ± 2 SD of the response to water delivery. Comparison of the number of cells responding to bile acids (expressed as a percentage of total responding cells per embryo) showed a 55% reduction (*P* = 0.01, Mann–Whitney *U* test) in mutant embryos while the number of cells responding to food odour was reduced by 38% (*P* = 0.04). The number of cells responding to amino acids and nucleotides showed no significant differences (Fig. 3e), demonstrating ift88 loss of function did not affect microvillus OSNs. Furthermore, examination of the mutant OSN population responses, at single cell resolution, revealed a 66% reduction in the amplitude of bile acid responses (*P* = 1.9E−08, Mann–Whitney *U* test; Fig. 3f and Additional file 1: Movies S3–S5) and a 37% reduction in the amplitude of food odour responses (*P* = 8.34E−11) compared to wild-type siblings (Fig. 3f, responding OSNs only). No significant differences in OSN-response amplitudes were observed after stimulation with amino acid (*P* = 0.10) or nucleotide mixture (*P* = 0.19) (Fig. 3f). We observed similar results when response amplitudes were averaged for all OSNs (responding and non-responding) (Additional file 1: Fig. S4 a, b). The absence of large changes in response latency or in the shape and strength of responses to amino acids and nucleotides in *ift88* mutant olfactory neurons (Additional file 1: Fig. S4 c, d) indicated that loss of cilia-driven fluid flow at the olfactory placode [34] was not a factor in our approach applying odourants directly to OSNs. Overall, the data show a consistent and significantly lower response of OSNs selectively to bile acids and food in the *ift88* mutants compared to the *ift88* wild-type siblings.

Reduction in OSN responses to odourants in *ift88* mutants is most likely due to observed ciliogenesis defects. Alternatively, loss of sensitivity could be due to reduced OSN survival as has been previously suggested [36]. We ruled out cell loss as a significant factor in reduced OSN responses by showing no loss of *omp* mRNA expression in *ift88* mutants up to 5 dpf (Fig. 3g, Additional file 1: Fig. S6a), no differences in mCherry expression in *Tg(omp:mCherry) ift88* mutants vs. siblings (Additional file 1: Fig. S6b), and no change in the presence of ciliated OSNs using anti-G_α/olf_/anti-GFP double immunofluorescence in *Tg(elavl3:GCaMP5) ift88* mutants in which the ciliated OSNs were identified by their characteristic flask-like shape (Fig. 3h, Additional file 1: Fig. S6c, arrows) [17]. As expected, we observed a severely reduced anti-G_α/olf_ signal in the ciliary region in the mutants (Fig. 3h’, Additional file 1: Fig. S3). Careful examination, however, revealed some residual anti-G_α/olf_ staining in the apical cell bodies of *ift88* mutant OSNs (Additional file 1: Figs. S3D, S5). Lastly, we observed no significant differences in the number of GCaMP5-positive OSNs per embryo used in the functional analyses (*P* = 0.10, Fig. 3i). We conclude that reduced OSN responses in *ift88* mutant olfactory placodes were due to a specific defect in ciliogenesis and not due to a reduction in the number of ciliated OSNs.

### *Ift172*-deficient embryos show reduced cilia-dependent OSN activity

**Fig. 4:**
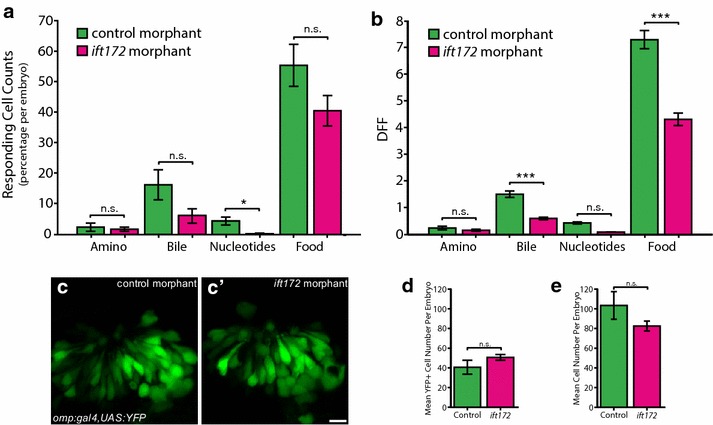
Effect of *ift172*-deficiency on cilia-dependent olfactory sensory neuron signalling in 2.5-dpf-old zebrafish. **a** Significant change in percentage of responding cells per embryo to nucleotides in *Tg(elavl3:GCaMP5) ift172* MO OSNs compared to control MO OSNs (*N* = 517 OSNs, 5 fish for control MO and *N* = 495 OSNs, 6 fish for *ift172* MO). **b** Significantly reduced response amplitude to bile acids and food odour in *Tg(elavl3:GCaMP5) ift172* MO responding OSNs compared to control MO responding OSNs. **c** No difference in Omp-positive OSNs between control and *ift172* MO *Tg(omp:gal4,UAS:YPF)* fish. **d** Quantification of C (*N* = 3 control MO, *N* = 4 *ift172* MO, *P* = 0.33). **e** No difference in number of GCaMP5-positive OSNs per embryo (*P* = 0.16). Bars represent mean and SEM (*P < 0.05, **P < 0.01, ***P < 0.001 Mann–Whitney *U* test for **a**, **b**, Student’s *t* test for **d**, **e**). Scale bar is 10 µm

To test for other Ift-deficiencies as potentially novel causes of hyposmia, we tested responses to odourants in *ift172*-deficient zebrafish. *ift172* morpholino knockdown was accomplished using a previously validated exon1 donor blocking MO [11] that caused retention of intron1 and complete loss of wild-type ift172 mRNA (Additional file 1: Fig. S2). *ift172* morphants phenocopied *ift172* mutants with ventral axis curvature, hydrocephalus and kidney cysts [37] (Additional file 1: Fig. S2). Similar to *ift88*-deficient embryos, analysis of six *Tg(elavl3:GCaMP5) ift172* morphants (495 OSNs) and five *Tg(elavl3:GCaMP5*) control morphants (517 OSNs) revealed a trend toward reduction in the number of cells responding to bile acids and food (Fig. 4a). Consistent with our results from *ift88* mutants, in *ift172*-deficient cells that responded to odourants, bile acid and food-response amplitudes were significantly reduced by 60% (*P* = 7.93E−8) and 41% (*P* = 3.89E−12), respectively. OSN-response amplitudes to amino acids (*P* = 0.38) and nucleotides (*P* = 0.11) in *ift172* morphants were not significantly changed (Fig. 4b, responding OSNs only). Similar results were observed when response amplitudes were averaged over all OSNs (responding and non-responding) except for nucleotides mixture where we observed a significantly lower response in the *ift172*-deficient embryos (44% reduction, *P* = 0.0018, Additional file 1: Fig. S4b). Loss of odourant sensitivity in *ift172* morphants was not due to OSN cell loss as similar numbers of ciliated OSNs were observed in *Tg(omp:gal4,UAS:YFP)* and *Tg(elavl3:GCaMP5)* control and morphant embryos (Fig. 4c–e). These results support and extend our data on *ift88* mutants and further, establish *ift172* deficiency as a novel genetic cause of hyposmia.

### Odour detection in 2.5 dpf *Tg(omp:GCaMP6)* zebrafish

**Fig. 5:**
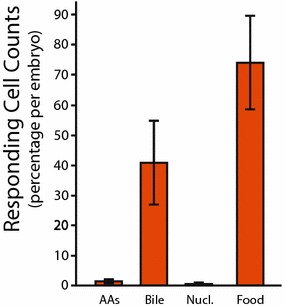
Odour detection in *Tg(omp:GCaMP6)* zebrafish at 2.5 dpf. Percentage of responding cells in *Tg(omp:GCaMP6)* fish after odour stimulation. Bars represent mean and SEM

To examine responses to odourants specifically in ciliated OSNs, we generated a *Tg(omp:GCaMP6)* line expressing GCaMP6 selectively in ciliated OSNs (Additional file 1: Fig. S8) and quantified the number of responding OSNs for different odourant classes at 2.5 dpf. The percentage of OSNs per embryo responding to amino acids and nucleotides was nearly undetectable in *Tg(omp:GCaMP6)* embryos while responses to bile acids and food were robust (Fig. 5). Our results differ from prior work [18] and show that as early as 2.5 dpf, GCaMP6 expressing, ciliated OSNs are bile acid selective.

## Discussion

The role of primary cilia as cellular signalling centres, coupling receptors to intracellular signal transduction, is now well established [1, 38]. However, studies of sensory cilia function have relied largely on indirect measurements of marker protein transport, cilia length or downstream molecular endpoints of cilia-associated signalling pathways in vitro [39–42]. Prior work using GCaMP readouts for in vivo cilia mechanosensation provided evidence for calcium elevation in response to cilia bending and mechanosensation in endothelial cells [43] and cilia of Kupffer’s vesicle, the zebrafish organ of laterality [44]. However, the concept that cilia bending is sufficient to propagate an intracellular calcium response has been challenged recently [45]. The function of photoreceptor cells in the retina depends on their sensory cilium outer segment [46]. Optokinetic responses (OKR) or electroretinograms [47, 48] can assay photoreceptor activity; however, these do not directly measure the activity of the ciliated cells since an altered OKR can also be caused by neuronal or muscle defects [49]. Direct patch-clamp of retinal cones has been performed in adult zebrafish retinas ex vivo [50]; however, the number of cells that can be studied per animal is small and this approach has not yet been demonstrated in larvae. Lateral line cilia in zebrafish have been used to study the role of dopamine in modulating mechanotransduction [51]. Most directly related to our work, DeMaria et al. demonstrated calcium responses to odourants in transgenic zebrafish larvae with GCaMP1.6 targeted to different OSNs classes, including ciliated OSNs [18]. Our work takes advantage of the accessibility of ciliated OSNs and a newer generation of transgenic GCaMP calcium indicators to probe in vivo requirements for quantitative assessment of sensory cilia signalling at the resolution of single cells.

The accessibility of OSN cilia facilitates stimulation with a broad range of distinct stimuli. We demonstrated a reduced bile acid response in Ift protein-deficient OSNs with severely reduced cilia length and number, while no change in amino acid response was observed. Consistent with these results, our analysis using the *Tg(omp:GCaMP6)* transgenic demonstrated that in zebrafish, bile acids but not amino acids are sensed by ciliated OSNs. In zebrafish, amino acids can be detected by microvillous OSNs [18]. Sensitivity of ciliated OSNs to bile acids has also been found in channel catfish and rainbow trout [52, 53]; however, in these species both ciliated and microvillus OSNs respond to amino acids, albeit using distinct signal transduction pathways (Galpha(olf)/cAMP vs. Galpha(q)/11/phospholipase C) [52]. In the marine fish Cabinza grunt, ciliated OSNs also respond to amino acids [54] indicating a degree of inter-species variation in OSN odourant specificity. In catfish, nucleotides are detected by microvillous OSNs [52]. Our results demonstrate no strong correlation between nucleotide odourant responses and ciliated OSNs, but should not be over-interpreted since at this stage of development there are a relatively small number of nucleotide responding OSNs compared to the populations responding to the other odourant classes. Other than developmental stage, this could be an effect of the specific nucleotides used in the odourant mixture. In our study, we observed a reduction in ciliated OSN activation similar to ex vivo results obtained in isolated *ift88* mutant mouse ciliated OSNs [7]. In this study, hypomorphic mutation of IFT88 leads to olfactory cilia loss and hyposmia as recorded ex vivo using electro-olfactograms [7]. We found in addition that *ift88*-deficient OSNs remained sensitive to amino acids demonstrating specificity of Ift defects. Our work demonstrates that odourant responses can be detected in vivo and that selective sensory deficiencies in the zebrafish *ift88* mutant and *ift172* morphants reveal a loss of sensory cilia function.

The absence of OSN cell loss in our studies of the *oval/ift88* mutant OSNs is in contrast to a prior report suggesting that ciliogenesis may be required for OSN viability [36]. This difference in results may be due to the use of DiI labelling, a classical assay of structural cilia defects in *C. elegans* [55], to detect OSNs, since the absence of OSN DiI labelling may simply reflect lack of cilia on otherwise viable cells. Also, the stage we chose to make measurements, 2.5 dpf, precedes the reported loss of ciliated OSNs in *oval* mutants [36]. Although responses to specific odourant classes were strongly reduced in Ift-deficient embryos, we detected some residual activity in the mutant/morphant OSNs. This may be caused by the persistence of a limited number of shortened olfactory cilia in the mutants (Fig. 3a, b; Additional file 1: Fig. S3b, d), which may be supported by maternally provided ciliary mRNAs and proteins in the zygotic *ift88* mutant [56]. It has been demonstrated that natural differences in cilia length in the olfactory epithelium in mice correlate with odour sensitivity, with shorter cilia being less sensitive to odours [57]. We demonstrated severely reduced anti-G_α/olf_ staining in the mutants, however, there was anti-G_α/olf_ signal from the cell body, indicating that even with reduced cilia, some odour detection might persist. Alternatively, detection of bile acids may not be exclusively dependent on ciliated OSNs at this early stage of development and could involve other classes of OSNs.

Our results demonstrate that ciliated OSNs are functional considerably earlier in development than previously shown (2.5 vs. 4 dpf [18]). This is significant since a key experimental advantage of the zebrafish for cilia proteome studies, transient gene knockdown (antisense, CRISPRi [58, 59]), is limited to early (1–3 dpf) developmental time windows. Our results demonstrating ciliated OSN signalling activity and impact of transient *ift172* knockdown in 2.5 dpf embryos support the use of this assay for specific sensory cilia mutants or drug screening and is consistent with prior studies of olfactory placode development. The zebrafish olfactory pit opens at 34–36 hpf and ciliated and microvillus OSNs can be observed by scanning EM at 48–50 hpf [33]. The first OSN projections to the presumptive olfactory bulb are present at 1 dpf [60] and olfactory bulb responses to the set of odourants we used have been recorded as early as 2.5 dpf [35]. Although we have not directly compared GCaMP1.6 [18] and GCaMP5 (this work), it is likely that the development of higher dynamic range biosensors will continue to improve selectivity and detection of cilia-dependent calcium signals in early embryos.

In addition to loss of cilia-dependent signalling in the *ift88* mutant, we demonstrate a sensory cilia signalling defect in *ift172*-deficient embryos. In humans, *IFT172* mutations cause isolated retinal degeneration, Jeune, Mainzer-Saldino and Bardet–Biedl syndromes [10–12]. Our study indicates that patients with *IFT7172* mutations may present with hyposmia as well. Reduced olfaction has been demonstrated in several ciliopathies, and could provide a non-invasive method to more easily diagnose ciliopathy patients [5, 61–63]. In preliminary experiments, we found no defects in cilia structure or protein localization in zebrafish *cep290* and *bbs4* Crispr/Cas9 mutants (data not shown) suggesting that for some ciliopathy genes, compensation mechanisms may mask ciliopathy phenotypes [64, 65]. Nonetheless, our demonstration of a novel hyposmia phenotype in *ift172*-deficient zebrafish highlights the value of measuring sensory cilia signalling functions directly in vivo and provides a new approach for future studies of cilia protein delivery and signalling as well as an additional starting point for the development of ciliopathy therapies [66].

## Conclusions

Calcium biosensor expression in ciliated, bile-sensitive zebrafish olfactory neurons can be used as a quantitative system for direct measurement of sensory cilia signalling in vivo in 2.5 dpf zebrafish larvae. Ift mutation and knockdown implicated *ift88* and *ift172*-deficiency in hyposmia, a reduced sense of smell. Our results highlight *ift172*-deficiency as a novel cause of hyposmia and support the idea that hyposmia can be used as a diagnostic indicator of ciliopathies.

## Additional file

**Additional file 1.** Additional material and methods, figures, figure legends, and legends for supplemental movies.

## Abbreviations

OSN: olfactory sensory neuron
ift: intraflagellar transport
OE: olfactory epithelium
BBS: Bardet Biedl syndrome
OKR: Optokinetic responses
