# Supplementary material for: Assaying sensory ciliopathies using calcium biosensor expression in zebrafish ciliated olfactory neurons
Source: Cilia. 2018 Mar 15;7:2. doi: 10.1186/s13630-018-0056-1 (PMC5856005; doi:10.1186/s13630-018-0056-1)
Supplement: Supplementary file 1 — Additional file 1. Additional material and methods, figures, figure legends, and legends for supplemental movies. [file 13630_2018_56_MOESM1_ESM.zip › Additional information.docx]

**ADDITIONAL DATA**

**Assaying sensory ciliopathies using calcium biosensor expression in zebrafish ciliated olfactory neurons**

Judith GM Bergboer1*, Cameron Wyatt2*, Christina Austin-­‐Tse1, Emre Yaksi2, 3, Iain A Drummond1*

**ADDITIONAL material and methods**

**Table S1. Primer sequences**

| **name** | **sequence (5' > 3')** |
| --- | --- |
| omp_FW | AATAAACAATACTG |
| omp_REV | TTGTTGTATTTTTAAC |
| ift172_MO_FW1 | ACGTTCTTTTACTGCCCTGAGTCTTAT |
| ift172_MO_REV1 | TCGATGCAGCCACAGTGAATTCCT |
| ift172_MO_FW2 | AGTATGCAGCTTAAGTACATGAAGACTCT |
| ift172_MO_REV2 | ATGTCTGAATCACGTTGCCCTCCTTT |

**ADDITIONAL MOVIE LEGENDS**

**Movie S1**

High-­‐speed video of moving motile cilia at the border of the olfactory pit in 2.5 dpf zebrafish. Movies were recorded at 200 frames per second (fps). Movie is playing at 15 fps.

**Movie S2**

Detection of food odor in OSNs of wild type *Tg(elavl3:GCaMP5)* zebrafish at 2.5 dpf. Movie was recorded in 3 fps, and is playing 11x faster. For clarity, movie colouring is changed from green into rainbow-­‐colours.

**Movie S3**

Detection of bile acids odor in OSNs of wild type *Tg(elavl3:GCaMP5)* zebrafish at 2.5 dpf. Movie was recorded in 3 fps, and played back at 25 fps. For demonstrating example responses, movie colouring is changed from green into rainbow-­‐colours. Both olfactory placodes are shown (laterally) along with GCaMP5 responses in forebrain neurons (center).

**Movie S4**

Loss of detection of bile acids odor in OSNs of oval/*ift88* mutant *Tg(elavl3:GCaMP5)* zebrafish at 2.5 dpf. Movie was recorded in 3 fps, and played back at 25 fps. For demonstrating example responses, movie colouring is changed from green into rainbow-­‐colours. Both olfactory placodes are shown (laterally) along with GCaMP5 responses in forebrain neurons (center).

**Movie S5**

Detection of food odor in OSNs of the same oval/*ift88* mutant *Tg(elavl3:GCaMP5)* zebrafish imaged in Movie S4. Movie was recorded in 3 fps, and played back at 25 fps. For demonstrating example responses, movie colouring is changed from green into rainbow-­‐colours. Both olfactory placodes are shown (laterally) along with GCaMP5 responses in forebrain neurons (center).

**ADDITIONAL Figure legends**

**Fig S1.** Severe reduction of IFT88 protein (anti-IFT88 antibody in red) expression is present in the cilia of the OE (marked by anti-acetylated tubulin staining) of the *ift88* mutant (B) compared to the *ift88* wildtype sibling (A). (C) Quantification of the signal in the red (IFT88) channel demonstrated a 99% decrease in intensity of the red staining. (N=3 fish per condition, P=5.5E-7, Student’s t-test). Bars represent mean and SEM. Scale bar is 10 µm.

**Fig S2.** (A) Phenotype of *ift172* MO fish, arrow points at kidney cyst. (B) Larger *ift172* PCR product using *ift172* cDNA as a template in *ift172* morphant. (C) Sequence analysis reveals that injection of *ift172* MO leads to retention of intron 1 in cDNA of morphants, leading to a predicted 16 amino acids peptide instead of the 1745 amino acids of the original protein.

**Fig S3.** Olfactory sensory cilia deficit in *oval/ift88* -/- mutants. (A) Olfactory sensory cilia in the center of a wild type olfactory placode stained with anti-Ift88 (red) and anti-acetylated tubulin (green; *cilia*) and imaged in confocal Z-stacks. Ift88 immunoreactivity was strongest in basal bodies (*bb's*). Scale bar in (A) equals 1 µm. (B) Wild type olfactory placode stained with anti-acetylated tubulin (green; cilia and neuronal cell body processes) and anti-cep290 (red; basal bodies). (C) *oval* mutant olfactory placode stained with anti-G_α/olf_ (red) and anti-acetylated tubulin (green) shows cilia loss with some short, residual G_α/olf_-positive axonemes (arrowheads). All panels are set at equivalent scale and represent a 3µm thick maximum intensity projection of the center of the olfactory placode.

**Fig S4.** Functional odor responses in 2.5 dpf fish. (A,B) Odor responses for all OSNs (both responding and non-responding) in analysis (A) *ift88* sibling and mutant and (B) control and *ift172* morphant. Most significances match well with those of responding cells only (Fig3f and 4b) offering further validation. Bars represent mean and SEM (*P<0.05, **P<0.01, ***P<0.001 Mann-Whitney U test). (C,D) Kinetics of averaged calcium responses to odorants in control and *ift88*-mutant olfactory placodes. (C,D) Kinetics of averaged calcium responses to odorants in control (C) and ift88 mutant (D) olfactory placodes. Control Tg(elavl3:GCaMP5) embryos (C) were imaged at 3 Hz during odorant exposure and individual cell responses were analyzed, averaged and quantified as change in flourescence divided by baseline fluorescence (ΔF/F). (n=554 neurons). ift88 mutant olfactory neuron responses to odorants (D) show a specific reduction in bile acid and food responses but no large change in reponse latency (n=376 neurons).

**Fig S5.** (A) Whole mount *in situ hybridization* using the *omp* probe showing *omp* expression in olfactory epithelia both in *ift88* mutants and wildtype siblings at 2, 3, 4, and 5 dpf. (B) *Tg(omp:mCherry)* fish demonstrate presence of Omp-positive OSNs in *ift88* wildtype siblings (B) and *ift88* mutants (B’) at 5 dpf. (C) Anti-GFP (green) and anti-G_α/olf_ (red) staining shows presence of ciliated OSNs in the *ift88* wildtype sibling, in the *ift88* mutant omp-positive cells are detected based on their characteristic flask like shape (arrows) in *Tg(elavl3:GCaMP5)* fish at 6 dpf. Scale bar is 50 µm in B, and 10 µm in C.

**Fig S6.** Ciliated OSNs stained with anti-GFP (green) and anti-G_α/olf_ (red) present in both sibling and *ift88* mutant. Lower panels: the anti-G_α/olf_ (red) signal only, demonstrating presence of anti-G_α/olf_ (red) staining in the cell bodies of both sibling and *ift88* mutant. Scale bar is 10 µm.

**Fig S7.** Anti-Cep290 antibody validation. In a 10 somite stage zebrafish embryo, the Cep290CT antibody (red) stain punctate structures at the base of Kuppfer’s vesicle cilia (anti-acetylated tubulin in green). Staining is lost when the antibodies are preincubated with antigen, demonstrating antibody specificity. Inset: magnified view of a single basal body region. Scale bar is 5 µm.

**Fig S8.** GCaMP6f expression in the olfactory placode in 2.5 dpf *Tg(omp:GCaMP6)* larvae. GCaMP6f in *Tg(omp:GCaMP6)* larvae was selectively expressed in flask shaped OSNs, representing a subset of ciliated OSNs adjacent to non-expressing (asterisk), presumably microvillus OSNs (see figure 1).
