# Supplementary figures and images for: Assaying sensory ciliopathies using calcium biosensor expression in zebrafish ciliated olfactory neurons
Source: Cilia. 2018 Mar 15;7:2. doi: 10.1186/s13630-018-0056-1 (PMC5856005; doi:10.1186/s13630-018-0056-1)

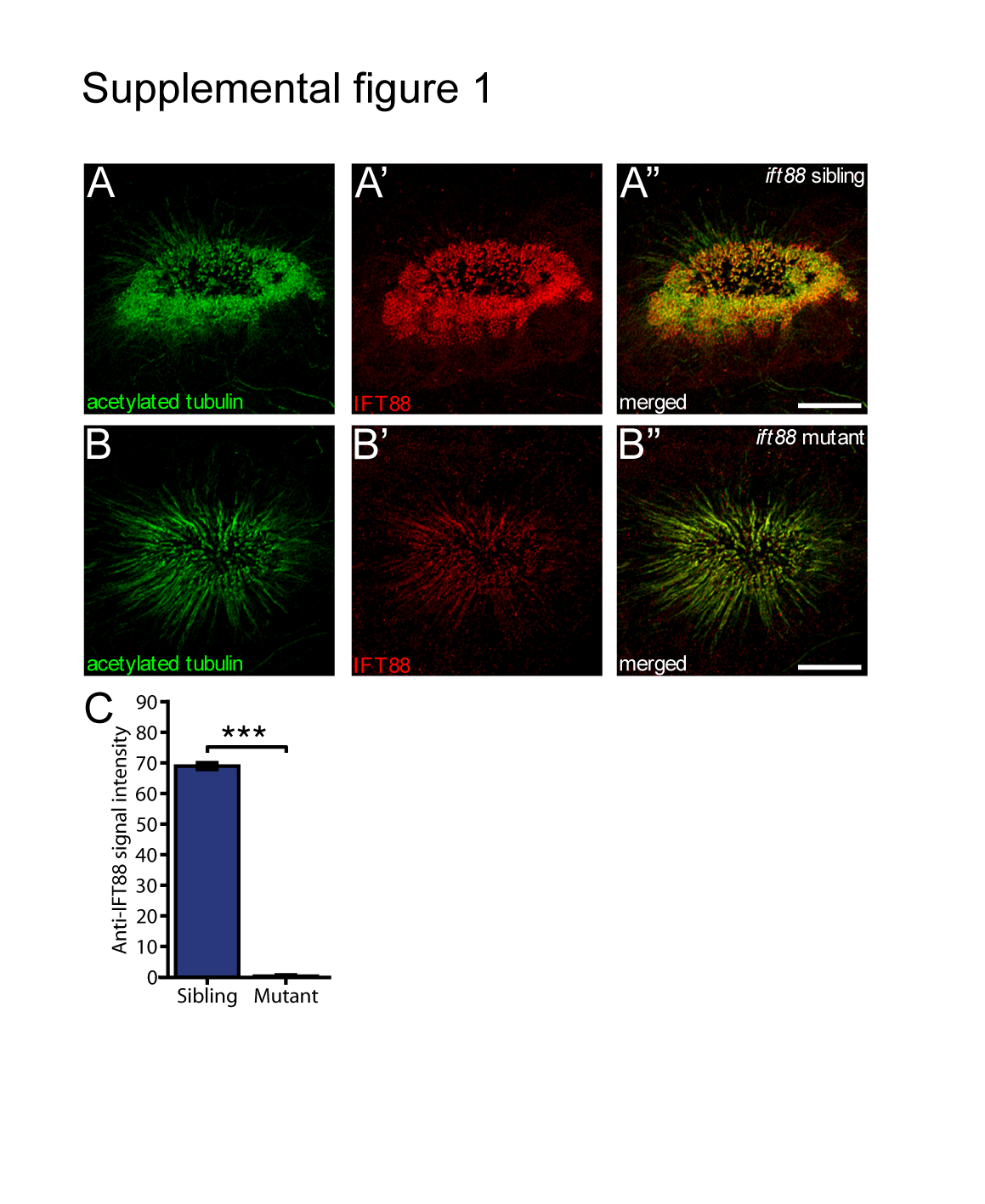

Supplement: Supplementary file 1 — Additional file 1. Additional material and methods, figures, figure legends, and legends for supplemental movies. [file 13630_2018_56_MOESM1_ESM.zip › FigS1.tif]

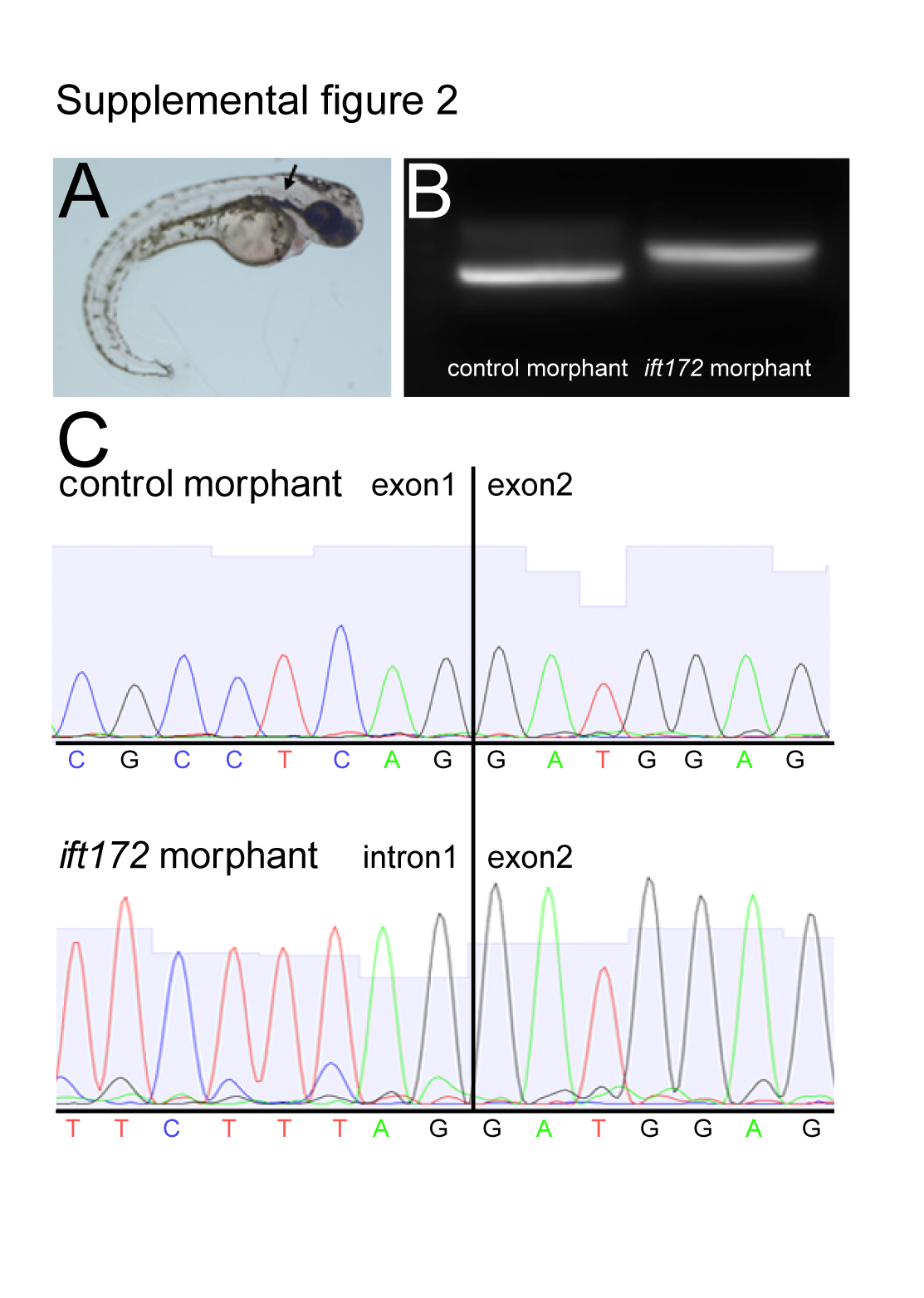

Supplement: Supplementary file 1 — Additional file 1. Additional material and methods, figures, figure legends, and legends for supplemental movies. [file 13630_2018_56_MOESM1_ESM.zip › FigS2.tif]

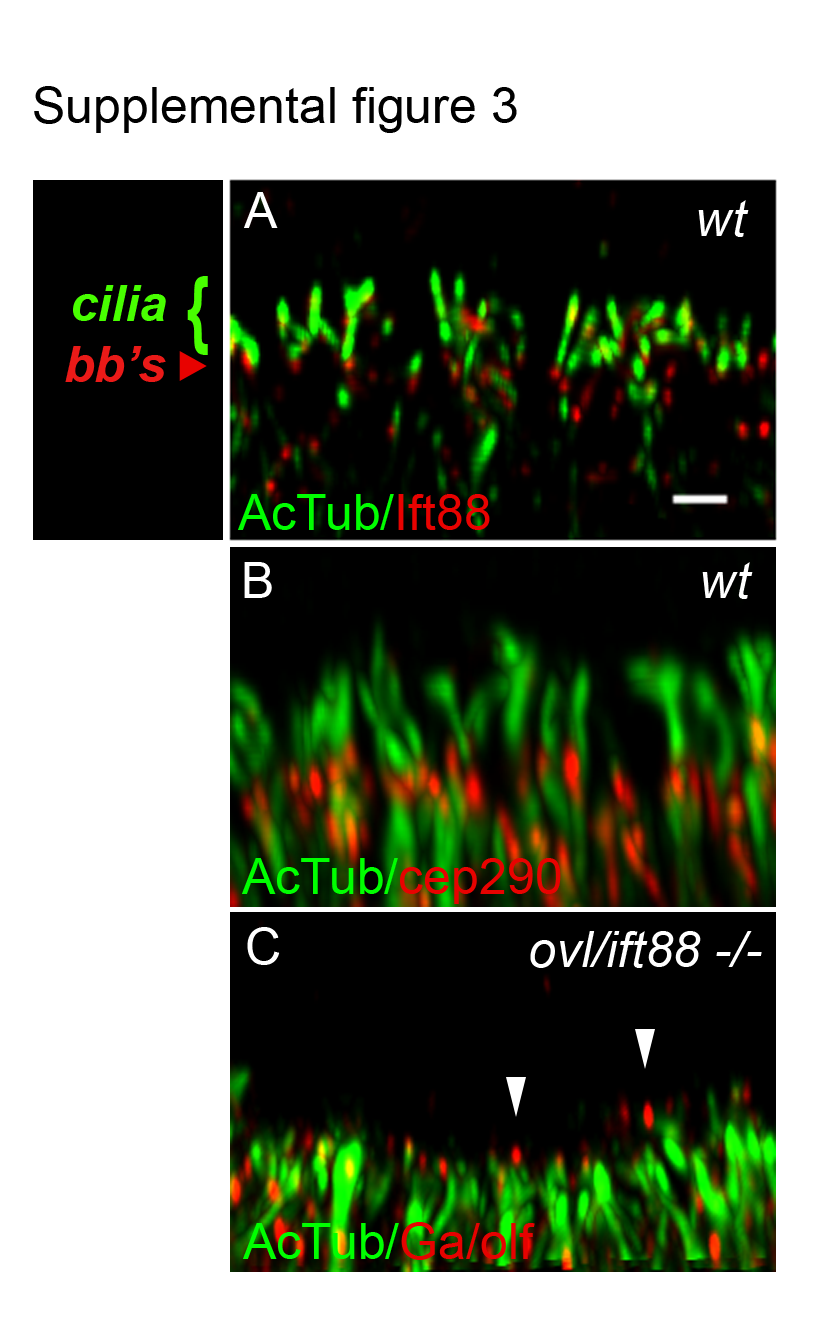

Supplement: Supplementary file 1 — Additional file 1. Additional material and methods, figures, figure legends, and legends for supplemental movies. [file 13630_2018_56_MOESM1_ESM.zip › FigS3.tif]

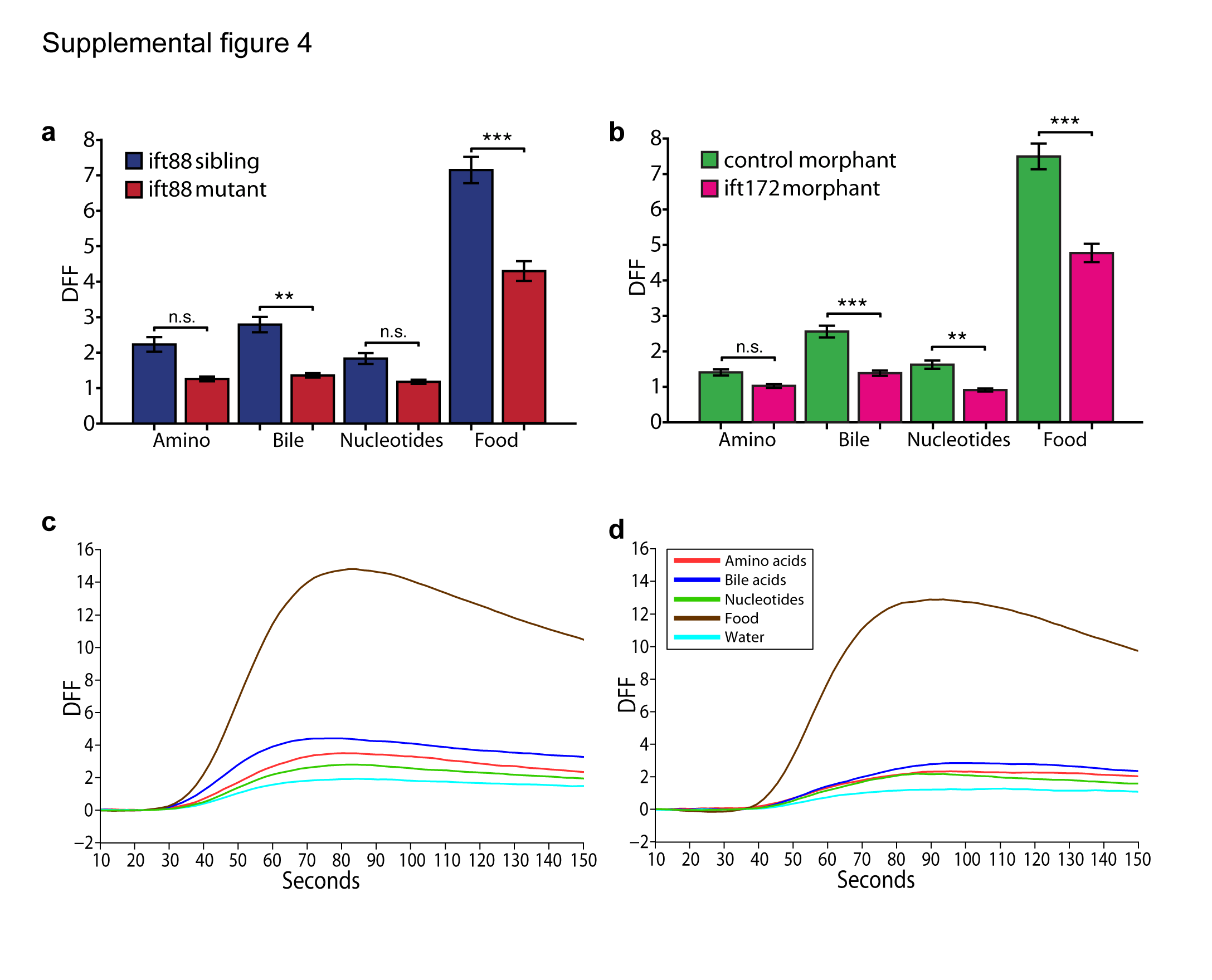

Supplement: Supplementary file 1 — Additional file 1. Additional material and methods, figures, figure legends, and legends for supplemental movies. [file 13630_2018_56_MOESM1_ESM.zip › FigS4.tif]

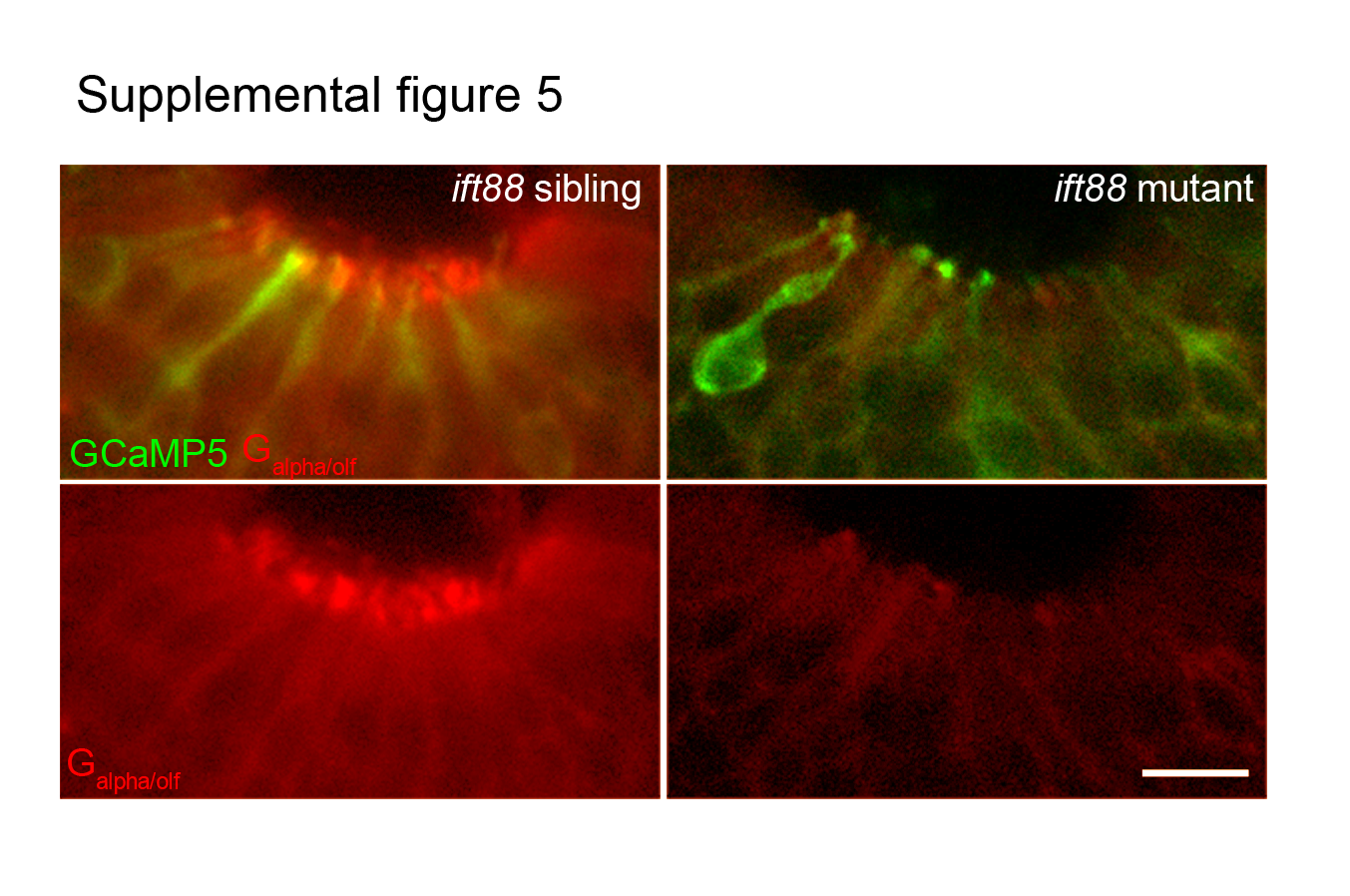

Supplement: Supplementary file 1 — Additional file 1. Additional material and methods, figures, figure legends, and legends for supplemental movies. [file 13630_2018_56_MOESM1_ESM.zip › FigS5.tif]

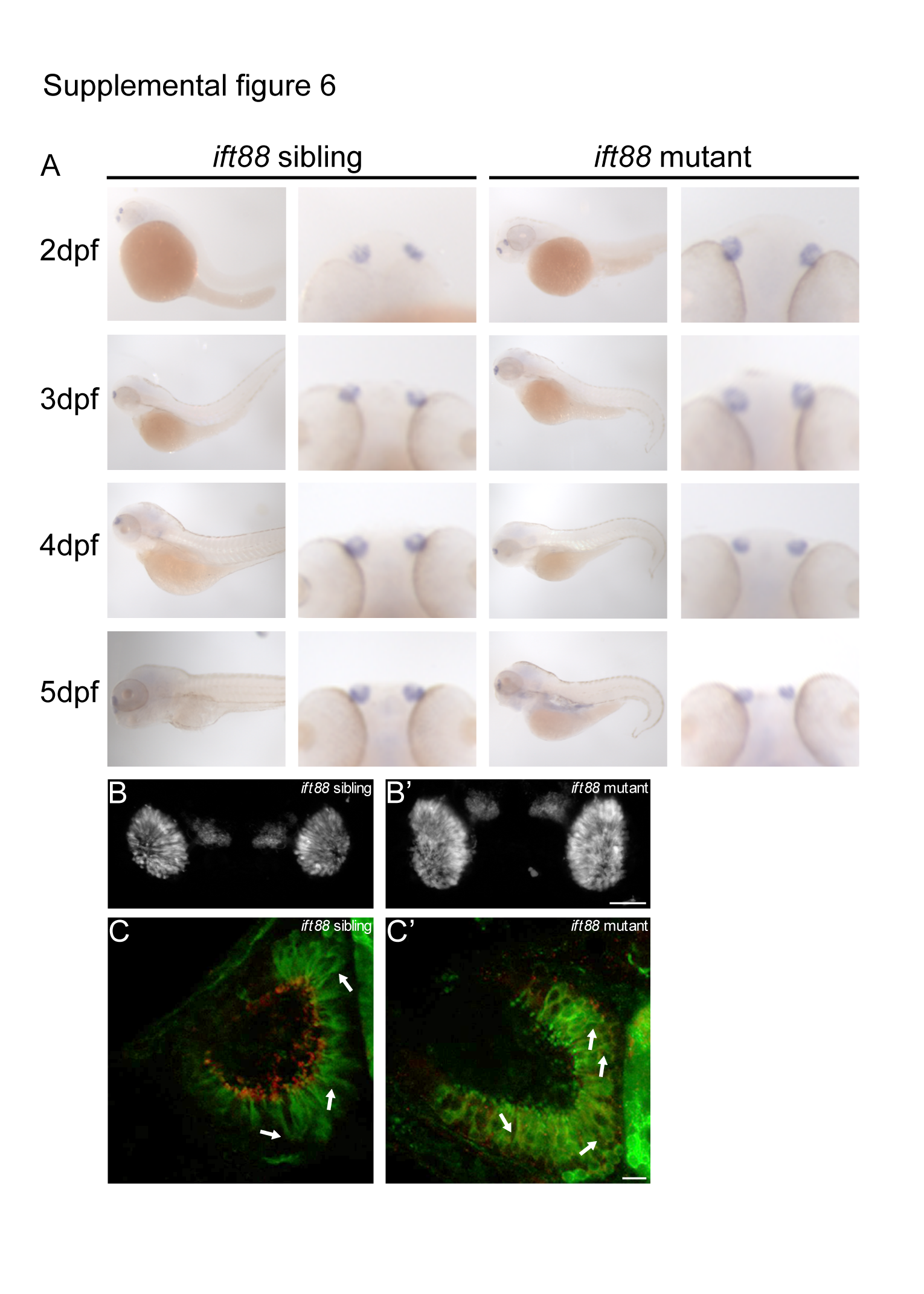

Supplement: Supplementary file 1 — Additional file 1. Additional material and methods, figures, figure legends, and legends for supplemental movies. [file 13630_2018_56_MOESM1_ESM.zip › FigS6.tif]

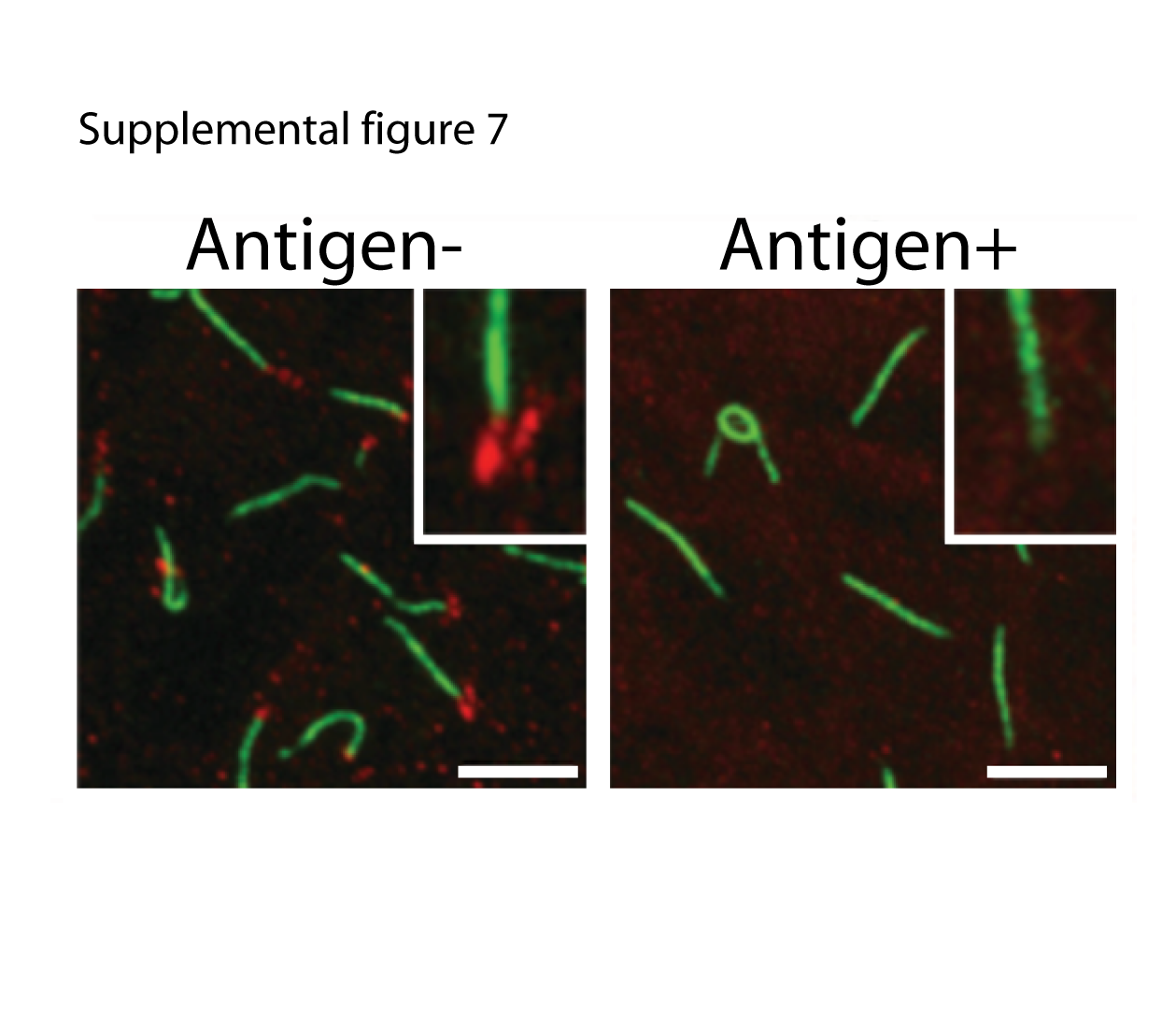

Supplement: Supplementary file 1 — Additional file 1. Additional material and methods, figures, figure legends, and legends for supplemental movies. [file 13630_2018_56_MOESM1_ESM.zip › FigS7.tif]

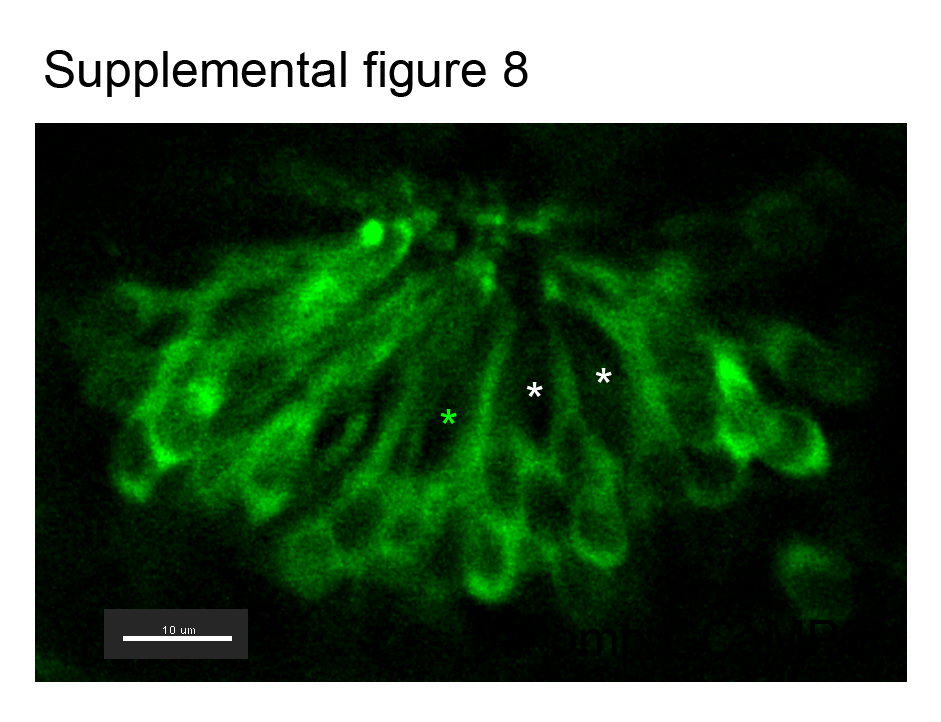

Supplement: Supplementary file 1 — Additional file 1. Additional material and methods, figures, figure legends, and legends for supplemental movies. [file 13630_2018_56_MOESM1_ESM.zip › FigS8.tif]
